# Perspectives on the psychological and emotional burden of having gestational diabetes amongst low-income women in Cape Town, South Africa

**DOI:** 10.1186/s12905-020-01093-4

**Published:** 2020-10-12

**Authors:** Lorrein Shamiso Muhwava, Katherine Murphy, Christina Zarowsky, Naomi Levitt

**Affiliations:** 1 Department of Medicine, Faculty of Health Sciences, University of Cape Town, Cape Town, South Africa; 2 Chronic Diseases Initiative for Africa (CDIA), Cape Town, South Africa; 3 Centre de recherche en santé publique (CReSP), Université de Montréal et CIUSSS du Centre-Sud-de-l’Île-de-Montréal and University of Montreal School of Public Health, Montreal, Canada; 4 School of Public Health, University of the Western Cape, Cape Town, South Africa

**Keywords:** Gestational diabetes mellitus, Type 2 diabetes, Pregnancy, Mental health, Low-income, South Africa

## Abstract

**Background:**

The diagnosis of gestational diabetes mellitus (GDM) may affect women’s mental wellbeing, functioning and quality of life, with potentially negative effects on treatment adherence. Identifying and addressing the psychological and emotional needs of women with GDM, could have benefits for sustainable long-term behavioural change following the affected pregnancy. This study explored the lived experiences of women with GDM and the impact of GDM on their experience of pregnancy and sense of well-being.

**Methods:**

Purposive sampling was used to recruit women who had been diagnosed with GDM in their previous pregnancy and received antenatal care at a tertiary hospital in Cape Town, South Africa. This was a descriptive qualitative study using a combination of focus groups and in-depth interviews for an in- depth exploration of women’s lived experiences of GDM, their context and perceived needs. Data analysis followed an iterative thematic analysis approach.

**Results:**

Thirty-five women participated in nine focus groups and five in-depth interviews. Women discussed the emotional and psychological burden of having GDM, highlighting (i) their initial emotional reactions to receiving a GDM diagnosis, (ii) their experience of adjusting to the constraints of living with GDM (iii) their feelings of apprehension about childbirth and their maternal role and (iv) their feelings of abandonment in the post-partum period once the intensive support from both health system and family ends.

**Conclusions:**

The current biomedical model used in the management of GDM, is highly foetal-centric and fails to acknowledge important psychological factors that contribute to women’s overall wellbeing and experience of pregnancy. These results demonstrate the importance of incorporating mental health support in the management and care for women with GDM in public health services, along with facilitating emotional support from partners and family members. Based on our findings, we recommend routine mental health and psychosocial vulnerability screening and monitoring for women diagnosed with GDM throughout pregnancy and postpartum to improve prognoses.

## Background

Gestational diabetes mellitus (GDM), currently defined as ‘diabetes diagnosed in the second or third trimester of pregnancy that is not clearly overt diabetes’ is one of the most common obstetric complications, affecting 18.4 million live births globally [1–3]. Women with GDM face increased risk of maternal, foetal and perinatal complications and have a significantly increased risk of developing type 2 diabetes [4, 5]. GDM may also have a negative impact on women’s mental wellbeing, functioning and quality of life [6–10]. The sparse literature on GDM and mental health suggests that the diagnosis of GDM is a risk factor for anxiety and stress during pregnancy [11–14], antenatal [15] and postpartum depression [16, 17], with potentially negative effects on treatment adherence [8, 9, 18]. The perceived stress of effectively managing GDM through lifestyle change coupled with the fear of failure to achieve glycaemic control may trigger depressive symptoms [19]. The management of GDM is centred on glycaemic control to prevent adverse obstetric outcomes [20–22], while the potential impact of the diagnosis on women’s psychological and emotional well-being is seldom considered in managing the condition.

In recognition of the psychological and longer term health needs of women with medical complications in pregnancy, who remain at risk after the pregnancy, the World Health Organisation (WHO) has called for the global health community to adopt a more comprehensive, woman-centred life course approach to maternal health that extends beyond pregnancy and childbirth [23, 24]. Understanding pregnant women’s lived experiences of morbidity as well as their psychological needs and concerns is important in itself. In low and middle-income countries (LMIC) settings such as South Africa, women may be exposed to high levels of stress due to numerous contextual factors [25]. Furthermore, as GDM may contribute to potential emotional disequilibrium during pregnancy [26], identifying and addressing the psychological and emotional needs of women with GDM, could also have benefits for sustainable long-term behavioural change following the affected pregnancy.

This study forms part of the formative research for an exploratory intervention trial with low-income women with GDM in South Africa, which aims to reduce their risk of progression to type 2 diabetes by providing continued support and care through the 12-month post-partum period. The purpose of this sub-study was to explore the lived experiences of women with GDM, including their experiences of antenatal and post-partum care and the impact of GDM on their lives, experience of pregnancy and sense of well-being.

## Methods

### Study design

This was a descriptive qualitative study [27], using a combination of focus groups and in-depth interviews to explore women’s lived experiences of GDM, their context and perceived needs. Focus groups enabled the collection of data on shared experiences and group norms around GDM in the context of a social group while individual in-depth interviews allowed exploration of the personal narratives of women in greater detail. The Consolidated criteria for Reporting Qualitative Research (COREQ) were followed in reporting the findings [28].

### Setting

The study site was a large tertiary academic teaching hospital in Cape Town, South Africa, which provides free health services in a context where the majority of the population cannot afford the cost of private health care. The hospital has a specialised antenatal clinic for women with diabetes in pregnancy (i.e.: Type I Diabetes, Type 2 Diabetes and GDM). Pregnant women are referred from various primary health care facilities around the Cape Town metropolitan district to receive antenatal care at this hospital. Antenatal care for women with GDM at this hospital is intensive, highly structured and follows local and international (i.e.; WHO and NICE) policy guidelines [21].

### Recruitment and data collection

Purposive sampling was used to recruit women who had been diagnosed with GDM in their previous pregnancy, according to the WHO 2013 criteria [29]. Women were eligible if they had received antenatal care including delivery at the hospital study site between 2014 and 2015 and were at least 1 year postpartum. Women with pre-existing diabetes or who had a stillbirth were excluded. Potential participants were identified from the medical records at the antenatal diabetes clinic in the hospital. Eligible participants were then contacted telephonically and invited to participate in the study.

Two female researchers (LSM & KM) with formal training in qualitative research methods conducted the focus groups and individual in-depth interviews in English, in a private venue on the hospital premises, away from the clinic. LSM has experience in public health research among low-income communities in South Africa. KM has extensive experience in qualitative research and has facilitated focus group discussions around sensitive topics. A diabetes nurse educator and counsellor, who was fluent in the local languages isiXhosa and Afrikaans, was present to assist participants who wanted to express themselves in their home language and to respond to specific questions around GDM management. A topic guide (Supplementary File 1) was used to structure the discussions to ensure that specific topics were covered consistently across focus groups. The discussions aimed to elicit women’s experiences of receiving a GDM diagnosis; antenatal care, including referral to the tertiary hospital; lifestyle modification; medication; delivery of the baby and postpartum health, as well as their opinions about how a potential intervention could meet the needs of women with GDM. Women were also encouraged to raise issues of importance to them and discuss these among themselves. This enabled certain topics to emerge which may otherwise not have been covered. Data collection continued until data saturation was reached after nine focus groups and no new information was emerging from the discussions. The focus groups were digitally recorded and transcribed verbatim.

### Data analysis

**Fig. 1:**
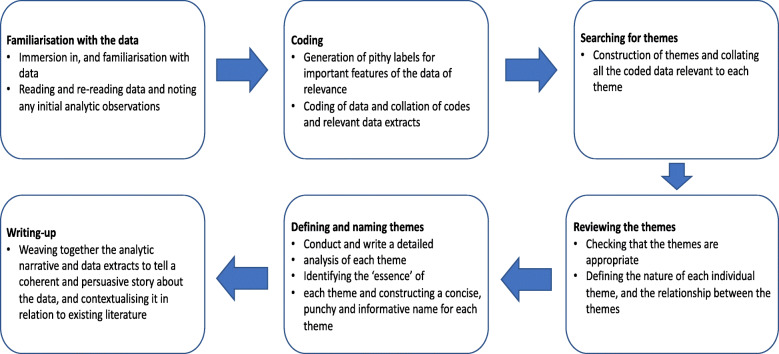
Flow chat of the six phases of thematic analysis. A summary of the six phases of the thematic analysis process as outlined in Braun et al. [30]

Data analysis followed an iterative thematic analysis approach [30, 31] summarised in Fig. 1 to explore the GDM journey through the lens of these women. Initial coding of the transcripts was done independently by two trained researchers as a measure to reduce the potential for researcher bias and increase rigour. The two researchers (LSM, KM) met regularly during the initial coding process to discuss their developing analysis, define codes and identify and resolve differences until consensus was reached on a common coding framework. This was then applied across the remaining transcripts. Codes were both inductively and deductively derived in that some codes were related to pre-determined concepts drawn from the literature and were present in the interview guide, whereas others emerged directly from issues raised by women as they spoke about their experiences. Feminist insights around maternal responsibility and mother-blaming (i.e. feeling blamed and maternal self-blame) provided some guidance for interpretation of the findings [32, 33].

## Results

The focus group discussions and individual in-depth interviews were completed over 3 months. Recruitment ceased after nine focus groups and five individual in-depth interviews with a total sample of 35 women, as data saturation had been reached. The participants’ ages ranged from 25 to 43 years old. Most of the women were married (60%) and unemployed (77%). The participants were of black African and ‘coloured’ (mixed ancestry) ethnicity and spoke mainly isiXhosa, Afrikaans and English. Our sample also included four foreign nationals who were conversant in English.

**Fig. 2:**
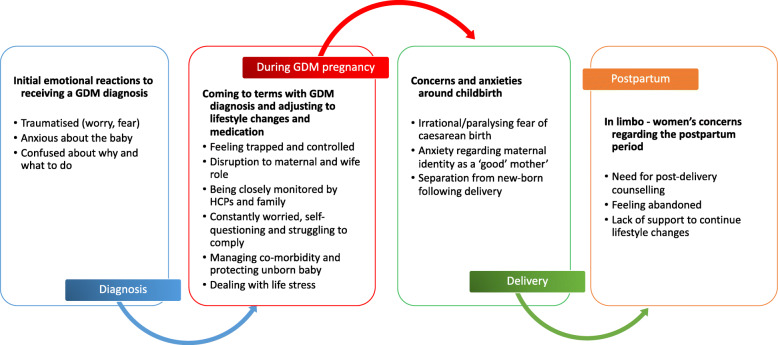
The different emotional burdens associated with the different phases of a GDM pregnancy. An overview of the sub-themes that relate to four different phases in the pregnancy affected by GDM

The interviews explored a range of issues related to GDM and several themes emerging from the data are reported elsewhere [34]. This paper focuses on the emotional and psychological aspects of women’s experience of GDM, which emerged as one of the most dominant topics. The findings are discussed under four sub-themes that relate to the four different stages in the pregnancy affected by GDM (see Fig. 2): (i) their initial emotional reactions to receiving a GDM diagnosis, (ii) their experience of adjusting to the constraints of living with GDM (iii) their feelings of apprehension about childbirth and their maternal role and (iv) their feelings of abandonment in the postpartum period once the intensive support from both the health system and family ends.

(I) “Why me?”: Shock and confusion at diagnosis

For virtually every woman interviewed, the diagnosis of GDM came as an unexpected shock. Reflecting back to the diagnosis during their second trimester, women described how at the time, they were unsure of the purpose of the blood tests and confused as to how or why they would have suddenly developed GDM.

*When I got here for my first visit, they said to me ‘You need to go to this floor, and they’ll see to you there.’ I didn’t understand why. I didn’t know what was happening. I just took the doctor’s instructions and went. They would take my blood, but I was never explained anything. (Focus Group 4, Participant 16).*

*I was unhappy because I was thinking I can’t be diabetic because in my family there’s no-one [with diabetes]. I was the first one. I was shocked. I didn’t know anything (Focus Group 8, Participant 28).*

One woman explained how she was so shocked to be told that her glucose was high that she refused to accept the diagnosis.

*They said, ‘Your sugar is high’. I said, ‘My sugar is high?!!I am not a diabetic, how can you say that my sugar is high?!!’ (Focus Group 2, Participant 8).*

Women felt disempowered by their lack of knowledge and this was a cause of much of their anxiety. They voiced their frustration with the lack of information from health care providers and the limited opportunity to discuss their GDM in detail. Some participants felt that health care providers did very little to calm their fears at diagnosis or to address their immediate concerns about it.

*You’re here because you need to undergo all these tests and things. They are just like; ‘We’ll review your case tomorrow’. They’re doing the best they can, but they’re not communicating it to you, and that’s what’s frustrating. (Focus Group 6, Participant 19).*

For some women, learning that there was something ‘wrong’ with the pregnancy, made them feel that they were failing to meet social expectations in their roles as women and mothers. This appeared particularly acute among women who were pregnant for the first time and more susceptible to feelings of failure:

*I was thinking what’s going to happen. I don’t want to lose my baby. It was my first baby, I didn’t know I could fall pregnant, so it was like a huge thing for me to just get it right the first time. (Focus Group 3, Participant 11)*

(II) “Feeling like a prisoner”: adjusting to the constraints of living with GDM

Most women gradually accepted their GDM diagnosis and began to implement health behaviour changes. However, they highlighted the burden placed on them by having to attend frequent antenatal care visits; the challenges of adhering to the strict, new healthier diet regimen; their fear of the results of the frequent blood glucose tests and the reaction this would elicit from the healthcare providers.

Women who had been hospitalised for close monitoring at some point during their pregnancy felt the loss of autonomy particularly acutely, describing how they felt oppressed by the hospital routine, the boredom, surveillance of the nurses and having to eat the bland ‘tasteless’ food. One woman analogized her experience of having GDM to being a prisoner, describing that she felt a loss of control over her own life and resented the constant monitoring and overbearing scrutiny by healthcare providers, a sentiment echoed by many other women.

*I would say I felt like a prisoner. I was never explained anything. I didn’t understand, even when I was here in hospital, they would say, ‘Come, you shouldn’t eat that’ that’s why I’m saying that I felt like a prisoner, because I didn’t know what was happening. No one was speaking to me (Focus Group 4, Participant 16).*

Women who were required to self-administer insulin as part of their management of GDM also felt particularly burdened by the experience.

*I didn’t know how to do the pricking at first, and they assumed that I did. There wasn’t room for me to ask questions, it was very quick, fast-paced. I was still in so much emotion; I couldn’t even recall how often she said I had to do it. (Focus Group 1, Participant 4).*

A few women reportedly rebelled against this loss of control by ‘cheating’ and secretly eating whatever they wanted to.

*I phoned my boyfriend to bring for me what I ask him to bring for me. I was always hiding food. The doctor and the nurse didn’t know that I was eating fish and chips and cool drinks.*^1^*(Focus Group 3, Participant 12).*

### Struggling to comply

Adjustment to having GDM was clearly an ongoing intricate process that required self-motivation, as well as social and professional support. All the women in our study reported being highly conscious of how their behaviour could affect their unborn baby, yet many admitted to struggling to balance adherence to healthier diet recommendations while managing comorbidities, maintaining relationships with family and health care providers as well as coping with other life stressors.

Each antenatal visit evoked fear and concern that by not always adhering to behaviour change recommendations, particularly regarding their diet, they may have caused harm to their unborn baby. One woman with a history of miscarriage described the fear she felt every time she was referred for an ultrasound that the doctor would find something wrong with the baby;

*I was going for ultrasound every time. For me it was like, just now they could tell me something negative, as was my experience with my previous baby where they told me something was wrong with baby. And now I was scared that I was going to hear something like that again. (Focus Group 5, Participant 17).*

### Managing co-morbidity

A few women in the study had pre-existing medical conditions such as hypertension and HIV, in addition to the GDM, which compounded their fears regarding their health and that of the baby. They also described the pressure they felt having to take a combination of medications, in addition to changing their health behaviour;

*I was just worried because I had high blood pressure with the diabetes, I was just worried like, is there going to be enough space for the baby to grow? Do I need to keep the sugar down, what am I going to do? How do I do it? It felt like a constant battle. (Focus Group 6, Participant 20).*

### Disruption to maternal and wife role

Being hospitalised and away from home disrupted families’ routines, as well as women’s maternal role and personal identity as the family’s caregiver. Even with sufficient social support from spouses and family members to assist with childcare at home, women were still often stressed and worried about the wellbeing of their children and expressed a sense of guilt for burdening their spouses with additional responsibilities.

*Now I think of the time when I was in hospital, the children were alone at home and my husband had to look for something for them to eat. Who was going to cook for them? (Focus Group 2, Participant 8).*

### Dealing with other life stressors

One woman, an immigrant from the Democratic Republic of Congo, described the stress of having to renew her documents at the immigration offices while she was hospitalised for poorly controlled GDM:

*I had to go to Home Affairs to renew my papers. I was already booked in the hospital because my sugar was high. The doctor wrote a letter to give to people at Home Affairs, because sometimes you can go, and they’re don’t see you. There are a lot of people and you can be there until late. (Focus Group 2, Participant 8).*

### Relationship with healthcare providers

Whilst several women reported positive experiences of being supported and encouraged by health care providers, a few women described how the nurses scolded and chastised them if their glucose was high. They believed that nurses had little sympathy for their difficulties in complying with the prescribed diet and resented being shamed for not meeting glucose targets and being treated like irresponsible children. Women also felt that nurses blamed them for having GDM because they were overweight:

*They make you feel like your body is failing. They say, ‘listen, you ate wrong and it’s because of you and, and it’s your fault if something happens to your baby.’ They need to educate the people, like, ‘listen, it’s a sickness, anybody can get it, but you can manage it’ (Focus Group 1, Participants 2)*

(III) Apprehension about birth and maternal role

Several women, particularly women in their first pregnancy, expressed a strong desire to have a “natural” birth and were frustrated and disappointed upon learning that they would deliver by caesarean section.

*I was in denial about having the caesarean, I was really fixed on having natural birth. It was my first child. I wanted the experience. (Focus Group 1, Participant 4).*

Others were afraid to undergo major surgery and initially protested when requested to consent to a caesarean birth;

*One of them [doctors] said ‘This is a big baby; you must deliver by C-section’. I have never had a Caesar in my life. I was shocked. I said’ I can’t sign this paper’ to say that they must take out the baby* via *Caesar. No! (Focus Group 2, Participant 8).*

Women also found the common practice of being separated from their babies immediately after the birth very stressful. Apparently, this was a necessary process in order to the baby to undergo blood glucose tests. However, women reported that this was not adequately explained to them, which left them feeling worried about what was happening to their baby and upset about being deprived of the opportunity to establish a bond with their new-born.

*The nurses went with the baby the whole day. I just saw the baby, and they took the baby to check the baby, maybe she also has sugar. So, you are not with the baby, the baby is in another place, do you know how that feels [gestures]? I started to stress; now where is my baby? (Focus Group 2, Participant 8)*

(IV) Feeling abandoned: The lack of post-partum follow-up and support

Following their discharge from hospital, the majority of women reported feeling a sense of great relief that they no longer had to worry about harming their baby, attend the hospital or follow the ‘diabetic diet’ and they looked forward to settling back into their “old lives”. Some women described how they felt a sense of abandonment when care abruptly ceased after delivery and there was no follow up contact.

*I had to figure it all out on my own afterwards, because there was no counselling or no doctor’s appointment that I could go to. I had to find my own way, basically … I lost all faith in public hospitals because of my experience and I was just discharged with no return letter to come for a check-up, or anything like that. (Interview 1, Participant 5).*

This state of being “in limbo” as a result of not having support from the health system once they were discharged from hospital was worsened by the diminished social support from partners and family members postpartum. One woman explained that while she made the financial sacrifices during pregnancy to eat healthy food, she could not afford to incur the extra expense postpartum:

*I know what I’m supposed to eat, but there are times I don’t have the food that I’m supposed to eat, then I have to eat whatever because I’m hungry; but I know what is good for me, but there are times I don’t have food. During pregnancy, I tried. I was motivated because I was scared for my unborn child, so I had to sacrifice for her. I didn’t want complications (Focus Group 7, Participant 22).*

In the aftermath of the GDM pregnancy, only a few women felt empowered to continue with the lifestyle changes made during pregnancy.

*During pregnancy your focus is your baby, because you want everything to be okay with baby. So, after baby is born, you take more time also into your own life again, and then you realise, okay, I have to focus on me now. If there’s someone also counselling you, and telling you look, this is what you need to do, that would be even better. (Interview 1, Participant 5).*

In a few cases, women felt that the experience of a GDM pregnancy had been a ‘wake-up call’ for them to adopt long-term lifestyle changes to prevent having GDM in subsequent pregnancies and avoid type 2 diabetes. They described having a renewed commitment to taking care of their health and concluded that the experience of GDM helped had helped to equip them to do this.

*I’m really more health wise, looking after myself, because it’s actually very important; because like I tell everybody, I do want another baby. That’s why I’m looking after myself (Focus Group 2, Participant 6).*

It is worth noting that several participants mistakenly interpreted their invitation to attend the focus group at the hospital as a form of follow up and an expression of care for their well-being, which they regarded in a very positive light. There was broad consensus that participation in the group discussion was both therapeutic and informative. Table 1 provides a summary of the results from the thematic analysis of women’s emotional and psychological experiences associated with a GDM pregnancy.

**Table 1:** Results from thematic analysis of women’s emotional and psychological experience of GDM by categories, codes and illustrative quotes

Categories/Sub-themes	Codes	Quotes
**Experience of GDM diagnosis:**		
Initial emotional reactions to receiving a GDM diagnosis	Traumatised	*It was very traumatising, and I cried for several days, because it was my first baby, and I waited so long for this baby. I was scared. (Focus Group 8, Participant 30)*
	Anxious about the baby	*For me it was scary, because I felt that I am going to lose my child. Or am I going to remain diabetic for the rest of my life, because I see people injecting themselves, I felt so bad, but the first thing was, is my child safe? My life changed completely (Focus Group 8, Participant 32)*
	Confused about why and what to do	*So, it really changed my life, I did not know it existed before I had it, that’s scary; if I could have known more about it, if I heard it somewhere. I really wish that I was informed, somehow. Even in our communities, it’s not spoken about, Gestational Diabetes. Maybe you could do something to prevent it from happening if you know information beforehand (Focus Group 1, Participant 4)*
**Experience of GDM pregnancy**		
Coming to terms with GDM diagnosis and adjusting to the constraints of living with GDM	Feeling trapped and controlled	*When I got here, I didn’t know where to go, the security told you must sit in that room. You’ll go sit there, you get to see, you go wee, go take your weight; do that, and after that you see the doctor and from there you go home. It’s like you’re not sitting with someone and they’re explaining to you, this is what’s happening. You don’t know, and that is a problem for me, because if you don’t know where you stand. (Focus Group 4, Participant 14)*
	Disruption to maternal and wife role	*Now I think of the time when I was in hospital, the children were alone at home and my husband had to look for something for them to eat. Who was going to cook for them? (Focus Group 2, Participant 8)*
	Being closely monitored	*I was seven and a half months, and they said they would have to book me inside [hospital] for a few months till I give birth to monitor. I couldn’t go home. And when I went home, I would only stay two days and then I will come back again; I had to make sure that for that days I’m at home, I must eat well but I was just telling myself, no one can tell me, but my boyfriend was always monitoring me. He didn’t even go to work, he gave up his job, because it was for his baby, his first child. (Focus Group 3, Participant 12)*
	Constantly worried, self-questioning and struggling to comply	*I was just worried like, is there going to be enough space for the baby to grow? Do I need to keep the sugar down, what am I going to do? How do I do it? It felt like a constant battle. (Focus Group 6, Participant 20)*
	Managing co-morbidity and protecting unborn baby	*In the process of booking for ANC, when they did all the tests, they found that I was HIV positive. My main worry during the pregnancy was of transmitting HIV to the baby (Focus Group 6, Participant 21)*
**Experience of GDM delivery**		
Feelings of apprehension about childbirth and their maternal role	Irrational/paralysing fear of caesarean birth	*My mother shared her experience with me of what she went though, and she had a Caesar because of the Diabetes, and I was scared I’m also going to have a Caesar, and she told me the things she went through, and I cried. I didn’t want to eat anything. I was so scared; it was very hard for me to accept. (Focus Group 8, Participant 26)*
	Anxiety regarding maternal identity as a ‘good’ mother’	*It was my first baby, I didn’t know I could fall pregnant, so it was, like, a huge thing for me to just get it right the first time. They also said they’re going to induce me. I said ‘I don’t actually want to be induced. I want the pain to come by itself (Focus Group 3, Participant 11)*
	Separation from new-born following delivery	*The nurses went with the baby the whole day. I just saw the baby, and they took the baby to check the baby, maybe she also has sugar. So, you are not with the baby, the baby is in another place, you know what you can feel [gestures]? I started to stress; now where is my baby? (Focus Group 2, Participant 8)*
**Post-partum period:**		
“In limbo” - feelings of abandonment once the intensive support from both the health system and family ends	Need for post-delivery counselling	*You will need to know what you’re going to have to do to maintain a healthy lifestyle, so if there’s counselling and someone to talk you through it and guide you, even better, especially for those women that are not knowledgeable, that don’t know these things, it’s very good to be counselled afterwards. (Interview 1, Participant 5)*
	Postpartum screening for diabetes	*You see, I keep on having excuses because I’m not sick that time. I just need to check. I don’t care enough, I would say, so, because I am not sick, I don’t see a need, which is wrong, but that’s what I do. You will only get worried when you got sick, then you start making time for those things (Interview 3, Participant 10)*

## Discussion

Our findings highlight the psychological distress experienced by women with GDM at different stages of the pregnancy. The GDM diagnosis triggered anxiety and stress for most women, echoing findings among women with GDM from high-income countries such as Australia, Canada and the United Kingdom [9, 15, 18, 19] and few LMIC countries such as Vietnam and China [13, 35]. However, there is a lack of studies on GDM and mental health from LMIC such as South Africa where maternal mental health remains a neglected area despite high prevalence of maternal depression and anxiety [25, 36]. Other reactions to the diagnosis of GDM by our participants such as denial or skepticism and a resistance to initiate the recommended lifestyle changes were as a result of women feeling ill-informed and disempowered about GDM. Lack of adequate information has also been highlighted as a major source of confusion, frustration and helplessness for women with GDM in other studies [13, 37, 38].

On a positive note, the majority of women in our study were able to overcome the initial shock and anxiety following the GDM diagnosis and gradually made the necessary adjustment to living with GDM. Their perceived anxiety did not increase as the pregnancy progressed suggesting that it could be reactive anxiety triggered by the unexpected diagnosis of GDM as opposed to intrinsic anxiety [18]. These findings are consistent with most of the literature which suggests that women are psychologically most vulnerable around the time of diagnosis [15, 22, 37]. However, our research also showed that the focus of women’s anxiety shifted as the pregnancy progressed to issues around compliance and the impending birth. Similarly, maternal distress relating to potential harm to the baby and obstetric complications continued up 37 weeks in more than half of a Canadian study population [19]. Understanding women’s emotional response to the GDM diagnosis is therefore important for designing appropriate interventions and improving their care during the remainder of their pregnancy [20].

A lack of capacity to adhere to recommendations and effectively control their glucose levels also contributed to heightened maternal distress in our study population. As we have reported previously [34], women experience several barriers to implementing lifestyle changes related to socio-economic status including food insecurity and inadequate social support. Psychosocial deprivation associated with low socio-economic status, affects women’s capacity for self- management and has been linked to poor prognosis among women with GDM in the UK and France [39, 40]. A US study among low-income mothers found that women who had diabetes in pregnancy (known diabetes and GDM) had nearly twice the risk of being diagnosed with depression during pregnancy or in the first year postpartum [41]. In our study, insulin administration, managing comorbidities and coping with other life stressors in addition to GDM were common sources of maternal distress associated with GDM, which have also been cited in other studies [37, 38, 42]. Having GDM was particularly burdensome for primigravidae women who felt ‘robbed’ of the joy of pregnancy. Interestingly, women with GDM in an Italian study reportedly experienced a better quality of life, compared to pregnant women without GDM [7] as a result of the improved health behaviours. Considering that among low-income women in South Africa, the experience of at least one stressful event can impact on maternal mental health [25], risk surveillance for mental health disorders, close monitoring and tailored programmes to reduce perceived emotional distress may have benefits for the overall well-being of women with GDM [10, 11, 16, 19].

An important finding in this study was the additional stress women felt having to manage the expectations of health care providers, partners, and family members. With a GDM pregnancy, maternal behaviour is heavily scrutinised, and women feel a sense of losing control over their bodies. There is an expectation of women diagnosed with GDM to make considerable adjustments to their health behaviour with little recognition of or sensitivity from the medical and social environment to the multiple barriers they face in changing these behaviours. For low-income women like those represented in our sample, the nature of the physical and social environment, availability and access to healthy affordable food and the extent of social support from partners and family all profoundly influence women’s capacity for lifestyle change and adherence to clinical recommendations [10, 22, 32, 33, 43]. Yet, women in our study and others report feeling that they are held primarily accountable for the health of their unborn child and that any pregnancy complications or adverse outcomes are perceived both by themselves and others as reproductive or maternal failure [22, 33, 44]. In South African society, as in many others, women’s identity is rooted in pregnancy and motherhood [45]. Self- blame is therefore common in women who experience a complicated pregnancy. This contributes significantly to heightened anxiety and an overwhelming sense of guilt, especially when maternal behaviour such as diet or smoking is implicated in the problem - a phenomenon described in feminist literature as mother- blaming [33].

Women in other studies have also reported feeling unsupported and isolated as a result of health care providers’ shaming and blaming attitudes [35, 37]. However, our study also shows that positive patient-provider relationships can create a platform for women with GDM to openly discuss their fears and concerns regarding their health with their health care providers. There is a need to re-frame messaging around developmental origins of health and disease to emphasise the role societal factors and not focus solely on the mothers [32]. Educating and engaging with partners and family members of women with GDM could afford them a clearer understanding of women’s needs and enable them to provide the GDM woman with appropriate social support [38].

Women with GDM are more likely to deliver by caesarean section compared to women without GDM [11]. The fear of delivery by caesarean section described by women in our study is consistent with the literature and has been reported as a major cause of anxiety during pregnancy among women in some high and low income countries [19, 46]. As reported in a recent systematic review, the increased risk of antepartum depression among women with GDM could partially be attributed to fear of obstetric complications and adverse outcomes for their health and that of their unborn baby [47]. The desire to experience a ‘normal’ vaginal birth and the perception of delivery by caesarean section as an indication of reproductive failure have also been cited as reasons for caesarean section refusal among women in Nigeria [46]. These fears and misconceptions could be addressed with proper counselling by health care providers to allay women’s fears.

The majority of our participants reported that they neither attended follow-up for diabetes screening nor sustained lifestyle change in the postpartum period. Pregnancy is an opportune time to promote long-term lifestyle changes to mitigate the risk of GDM in future pregnancies and the subsequent risk of developing type 2 diabetes [37]. Yet, based on their experiences during the GDM pregnancy, the postpartum period signified relief and freedom to resume unhealthy behaviours. According to a recent systematic review, women’s postpartum behavior is influenced by their perception of risk for diabetes as well as other factors such as affordability and fragmented health systems [48]. The low perception of future risk for type 2 diabetes evident in our study could be attributed to the highly foetal-centric approach to managing GDM in our context. Behaviour change recommendations are framed around foetal health and focused on blood glucose monitoring to prevent adverse pregnancy outcomes. As a result, without continued psychosocial support from health services and family in the postpartum period, women feel abandoned and *in limbo*, which hinders their ability to maintain health behaviour changes. Finnish and American studies have found a significant association between GDM and postnatal depression using the validated Edinburgh Postnatal Depression Scale [11, 49]. Only one woman in our study reported having postnatal depression following the GDM pregnancy and similar studies from LMIC are still lacking. Postnatal depression may interfere with women’s capability and motivation to engage in health behaviour changes [34], further affirming the importance of postnatal follow up, screening and counselling for women with GDM [37, 49].

### Strengths and limitations

Our findings add to the much-needed body of literature on maternal mental health in resource constrained settings facing complex burdens of disease such as South Africa and provide a basis for future studies. To the best of our knowledge, this is the first study in Africa to explore the impact of GDM on women’s experience of pregnancy and sense of well-being. Assessment of maternal distress (including anxiety and stress) was based on women’s self-reported experiences rather than a validated screening tool, given that this study aimed to gain an in-depth understanding of women’s lived experiences and the psychological impact of GDM on their lives. Future research should assess extent of maternal distress among women diagnosed with GDM using validated instruments such as the Edinburgh Postnatal Depression Scale. Generalizability of the findings to all women with GDM is limited due to the qualitative study design. Women’s perspectives may have been influenced by recall bias as they were at least 1 year postpartum at recruitment. However, the time elapsed between the pregnancy and inclusion in the study may have been sufficient for women to reflect on their experiences. Lastly, our participants were identified from women with GDM who had attended antenatal care at one of two large tertiary referral hospitals in the province. Based on the diversity of the sample, our participants are somewhat demographically representative of women who utilize these health services.

## Conclusions

South Africa has made steady progress over the last decade to improve maternal health services and reduce maternal mortality through public health programmes and initiatives (e.g.; Prevention of Mother to Child Transmissions (PMTCT)). Yet attention to psychological distress and the provision of maternal mental health services for women with complicated pregnancies such as GDM remains a neglected area. The current biomedical model used in the management of GDM, is highly foetal-centric and fails to acknowledge important psychological factors that contribute to women’s overall wellbeing and experience of pregnancy [21, 50]. Our findings echo prevalent views around the ‘burden’ of maternal responsibility and the culture of mother-blaming. Acknowledgement of other contributing factors such as the prevailing physical and social environment [34] is a crucial step towards shared accountability and provision of appropriate support for women with GDM. The study findings have several implications for the management of GDM in South Africa. Health policy makers and health care providers should recognize the impact of a GDM- disrupted pregnancy [51] on women’s mental health, emotional wellbeing and quality of life and how the added psychological stress may affect adherence with treatment and recommended lifestyle changes [18]. Based on these findings, we recommend routine mental health and psychosocial vulnerability screening and monitoring for women diagnosed with GDM throughout pregnancy and postpartum [9, 41] to improve prognoses. Furthermore, these results demonstrate the importance of incorporating mental health support in health policies and clinical practice for the management and care for women with GDM in public health services, along with facilitating emotional support from partners and family members.

## Supplementary information

**Additional file 1: Supplementary File 1.** Topic guide for focus groups and in-depth interviews. A topic guide (Supplementary File 1) was used to structure the discussions and to ensure that specific topics were covered consistently across focus groups.

## Data Availability

The datasets analysed during the current study are not publicly available to preserve participant anonymity.

## Abbreviations

GDM: Gestational Diabetes Mellitus
WHO: World Health Organisation
LMIC: Low and middle-income countries
COREQ: Consolidated criteria for Reporting Qualitative Research
PMTCT: Prevention of Mother to Child Transmissions

## Footnotes

1 A term used to describe sweetened carbonated beverages
